# Entropy Multiparticle Correlation Expansion for a Crystal

**DOI:** 10.3390/e22091024

**Published:** 2020-09-13

**Authors:** Santi Prestipino, Paolo V. Giaquinta

**Affiliations:** Dipartimento di Scienze Matematiche ed Informatiche, Scienze Fisiche e Scienze della Terra, Università degli Studi di Messina, Viale F. Stagno d’Alcontres 31, 98166 Messina, Italy; paolo.giaquinta@unime.it

**Keywords:** entropy multiparticle correlation expansion, one- and two-body density functions, one- and two-body entropy

## Abstract

As first shown by H. S. Green in 1952, the entropy of a classical fluid of identical particles can be written as a sum of many-particle contributions, each of them being a distinctive functional of all spatial distribution functions up to a given order. By revisiting the combinatorial derivation of the entropy formula, we argue that a similar correlation expansion holds for the entropy of a crystalline system. We discuss how one- and two-body entropies scale with the size of the crystal, and provide fresh numerical data to check the expectation, grounded in theoretical arguments, that both entropies are extensive quantities.

## 1. Introduction

The entropy multiparticle correlation expansion (MPCE) is an elegant statistical-mechanical formula that entails the possibility of reconstructing the total entropy of a many-particle system term by term, including at each step of summation the integrated contribution from spatial correlations between a specified number of particles.

The original derivation of the entropy MPCE is found in a book by H. S. Green (1952) [1]. Green’s expansion applies for the canonical ensemble (CE). In 1958, Nettleton and M. S. Green derived an apparently different expansion valid in the grand-canonical ensemble (GCE) [2]. It took the ingenuity of Baranyai and Evans to realize, in 1989, that the CE expansion can indeed be reshuffled in such a way as to become formally equivalent to the GCE expansion [3].

A decisive step forward was eventually taken by Schlijper [4] and An [5], who have highlighted the similarity of the entropy formula to a cumulant expansion, and the close relationship with the cluster variation method (see, e.g., [6]). Other papers wherein in various ways the combinatorial content of the entropy MPCE is emphasized are references [7,8,9,10].

Since the very beginning it has been clear that the successive terms in the entropy expansion for a homogeneous fluid are not all of equal importance. In particular, the contributions from correlations between more than two particles are only sizable at moderate and higher densities. However, while the two-body entropy is easily accessed in a simulation, computing the higher-order entropy terms is a prohibitive task (see, however, reference [11]). Hence, the only viable method to compute the total entropy in a simulation remains thermodynamic integration (see e.g., [12]). The practical interest for the entropy expansion has thus shifted towards the residual multiparticle entropy (RMPE), defined as the difference between excess entropy and two-body entropy. The RMPE is a measure of the impact of non-pair multiparticle correlations on the entropy of the fluid. For hard spheres, Giaquinta and Giunta have observed that the RMPE changes sign from negative to positive very close to freezing [13]. At low densities the RMPE is negative, reflecting a global reduction (largely driven by two-body correlations) of the phase space available to the system as compared to the ideal gas. The change of sign of the RMPE close to freezing indicates that fluid particles, which at high enough densities are forced by more stringent packing constraints, start exploring, this time in a cooperative way, a different structural condition on a local scale, preluding to crystallization on a global scale. Since the original observation in [13], a clear correspondence between the RMPE zero and the ultimate threshold for spatial homogeneity in the system has been found in many simple and complex fluids [14,15,16,17,18,19,20,21,22,23,24], thereby leading to the belief that the vanishing of the RMPE is a signature of an impending structural or thermodynamic transition of the system from a less ordered to a more spatially organized condition (freezing is just an example of many). Albeit empirical, this entropic criterion is a valid alternative to the far more demanding exact free-energy methods when a rough estimate of the transition point is deemed sufficient. For a simple discussion of the interplay between entropy and ordering, the reader is referred to reference [25]; see instead references [26,27] for general considerations about the entropy of disordered solids.

A pertinent question to ask is, what happens to the RMPE on the solid side of the phase boundary, considering that an entropy expansion also holds for the crystal? This is precisely the problem addressed in this paper. Can the scope of the entropic criterion be extended in such a way that it also applies for melting? As it turns out, we can offer no definite answer to this question, since theory alone does not go far enough and we ran into a serious computational bottleneck: while the formulae are clear and the numerical procedure is straightforward, it is extremely hard to obtain reliable data for the two-body entropy of a three-dimensional crystal. We have only carried out a limited test on a triangular crystal of hard disks, but our results are affected by finite-size artifacts that make them inconclusive. Nevertheless, a few firm points have been established: (1) the approximate entropy expressions obtained by truncating the MPCE at a given order can all be derived from an explicit functional of the correlation functions up to that order; (2) the one-body entropy for a crystal is an extensive quantity (the same is held to be true for the two-body entropy, but our arguments are not sufficient for a proof); (3) the peaks present in the crystal one-body density have a nearly Gaussian shape; (4) we have also clarified the role of lattice symmetries in dictating the structure of the two-body density, which is explicitly determined at zero temperature.

This paper is organized as follows. In Section 2 we resume the formalism of the entropy expansion for homogeneous fluids and provide the basic tools needed for its extension to crystals. Then, in Section 3 we exploit the symmetries of one- and two-body density functions to predict the scaling of one- and two-body entropies with the size of the crystal. The final Section 4 is reserved to concluding remarks.

## 2. Derivation of the Entropy MPCE

In this Section, we collect a number of well-established results on the entropy MPCE, with the only purpose of setting the language and notation for the rest of the paper. First, we recall the derivation of the entropy formula for a one-component system of classical particles in the canonical ensemble. Such an ensemble choice is by no means restrictive, since, as we show next, it is always possible to take advantage of the sum rules obeyed by the canonical correlation functions to arrange the entropy MPCE in an ensemble-invariant form. Then, in the following Section we present an application of the formalism to crystals.

The canonical partition function of a system of *N* classical particles of mass *m* at temperature *T* is $Z_{N}=Z_{N}^{\mathrm{id}}Z_{N}^{\mathrm{exc}}$, where the ideal and excess parts are given by

(1) $$Z_{N}^{\mathrm{id}}=\frac{1}{N!}{\left(\frac{V}{\Lambda^{3}}\right)}^{N}\;\;\;\;\;\;\mathrm{and}\;\;\;\;\;\;Z_{N}^{\mathrm{exc}}=\frac{1}{V^{N}}\int d^{3}R_{1}\cdots d^{3}R_{N}\;e^{-\beta U(R^{N})}\;.$$

In Equation (1), *V* is the system volume, $\beta =1/(k_{B}T)$, $\Lambda =h/\sqrt{2\pi mk_{B}T}$ is the thermal wavelength, and $U(R^{N})$ is an arbitrary potential energy. As the particles are identical, for each n=1,2,…,N the cumulative sum of all *n*-body terms in *U* is invariant under permutations of particle coordinates (we can also say that *U* is $S_{N}$-invariant, $S_{N}$ being the symmetric group of the permutations on *N* symbols). The CE average of a function *f* of coordinates reads

(2) $$\langle f(R^{N})\rangle \equiv \frac{1}{V^{N}}\int d^{3}R_{1}\cdots d^{3}R_{N}\;f\left(R^{N}\right)\pi_{\mathrm{can}}\left(R^{N}\right)\;\;\;\;\;\;\mathrm{with}\;\;\;\;\;\;\pi_{\mathrm{can}}\left(R^{N}\right)=\frac{e^{-\beta U(R^{N})}}{Z_{N}^{\mathrm{exc}}}\;,$$

where $\pi_{\mathrm{can}}\left(R^{N}\right)$ is the configurational part of the canonical density function. Finally, the excess entropy $S_{N}^{\mathrm{exc}}\equiv S_{N}-S_{N}^{\mathrm{id}}$ reads

(3) $$\frac{S_{N}^{\mathrm{exc}}}{k_{B}}=-\frac{1}{V^{N}}\int d^{3}R_{1}\cdots d^{3}R_{N}\;\pi_{\mathrm{can}}\left(R^{N}\right)\ln\pi_{\mathrm{can}}\left(R^{N}\right)=-\langle \ln\pi_{\mathrm{can}}\left(R^{N}\right)\rangle \;.$$

We define a set of marginal density functions (MDFs) by

(4) $$\begin{array}{ccc} P^{\left(N\right)}\left(R^{N}\right) & = & \pi_{\mathrm{can}}\left(R^{N}\right)\;;\\ P^{\left(n\right)}\left(R^{n}\right) & = & \frac{1}{V^{N-n}}\int d^{3}R_{n+1}\cdots d^{3}R_{N}\;\pi_{\mathrm{can}}\left(R^{N}\right)\;\;\;\;\;\;\left(n=1,\ldots ,N-1\right)\;. \end{array}$$

Owing to $S_{N}$-invariance of $\pi_{\mathrm{can}}$, it makes no difference which vector radii are integrated out in Equation (4); hence, $P^{\left(n\right)}\left(r^{n}\right)$ is $S_{n}$-invariant (for example, $P^{\left(2\right)}\left(r,r^{\prime }\right)=P^{\left(2\right)}\left(r^{\prime },r\right)$). The following properties are obvious:

(5) $$\frac{1}{V^{n}}\int d^{3}R_{1}\cdots d^{3}R_{n}\;P^{\left(n\right)}\left(R^{n}\right)=1\;\;\;\;\;\;\mathrm{and}\;\;\;\;\;\;\frac{1}{V}\int d^{3}R_{n+1}\;P^{\left(n+1\right)}\left(R^{n+1}\right)=P^{\left(n\right)}\left(R^{n}\right)\;.$$

Then, the *n*-body density functions (DFs), for n=1,…,N, can be expressed as

(6) $$\rho^{\left(n\right)}\left(r^{n}\right)\equiv \langle \underset{i_{1}\ldots i_{n}}{\sum^{\prime }}\delta^{3}\left(R_{i_{1}}-r_{1}\right)\cdots \delta^{3}\left(R_{i_{n}}-r_{n}\right)\rangle =\frac{N!}{(N-n)!}\frac{P^{\left(n\right)}\left(r^{n}\right)}{V^{n}}\;,$$

where the sum in (6) is carried out over all *n*-tuples of distinct particles (for example, the sum for n=2 contains N(N−1) terms). We note that $P^{\left(1\right)}=1$ and $\rho^{\left(1\right)}=N/V\equiv \rho$ if no one-body term is present in *U*, i.e., if no external potential acts on the particles (then *U* is translationally invariant). $P^{\left(1\right)}\left(r\right)/V$ is the probability density of finding a particle in r; hence, $\rho^{\left(1\right)}\left(r\right)=NP^{\left(1\right)}\left(r\right)/V$ is the number density at r. Similarly, $P^{\left(2\right)}\left(r,r^{\prime }\right)/V^{2}$ is the probability density of finding one particle in r and another particle in $r^{\prime }$; hence, $\rho^{\left(2\right)}\left(r,r^{\prime }\right)=N\left(N-1\right)P^{\left(2\right)}\left(r,r^{\prime }\right)/V^{2}$ is the density of the number of particle pairs at $\left(r,r^{\prime }\right)$. As $r^{\prime }$ increasingly departs from r, the positions of two particles become less and less correlated, until $P^{\left(2\right)}\left(r,r^{\prime }\right)=P^{\left(1\right)}\left(r\right)P^{\left(1\right)}\left(r^{\prime }\right)$ at infinite distance. We stress that this cluster property holds in full generality, even for a broken-symmetry phase.

The *n*-body reduced density functions, for n=2,…,N, read

(7) $$\begin{array}{ccc} & & g^{\left(n\right)}\left(r^{n}\right)\equiv \frac{\rho^{\left(n\right)}\left(r^{n}\right)}{\rho^{\left(1\right)}\left(r_{1}\right)\cdots \rho^{\left(1\right)}\left(r_{n}\right)}=\left(1-\frac{1}{N}\right)\cdots \left(1-\frac{n-1}{N}\right)Q^{\left(n\right)}\left(r^{n}\right)\\ & & \mathrm{with}\;\;\;\;\;\;Q^{\left(n\right)}\left(r^{n}\right)=\frac{P^{\left(n\right)}\left(r^{n}\right)}{P^{\left(1\right)}\left(r_{1}\right)\cdots P^{\left(1\right)}\left(r_{n}\right)}\;. \end{array}$$

These functions fulfill the property

(8) $$\frac{1}{V}\int d^{3}R_{n+1}\;P^{\left(1\right)}\left(R_{n+1}\right)g^{\left(n+1\right)}\left(R^{n+1}\right)=\left(1-\frac{n}{N}\right)g^{\left(n\right)}\left(R^{n}\right)\;,$$

which also holds for n=1 if we define $g^{\left(1\right)}\equiv 1$. For a homogeneous fluid, $g^{\left(2\right)}\left(r,r^{\prime }\right)=g(|r-r^{\prime }\left|\right)$. From now on, we adopt the shorthand notation $P_{12\ldots n}=P^{\left(n\right)}\left(R^{n}\right)$ and $Q_{12\ldots n}=Q^{\left(n\right)}\left(R^{n}\right)$. Moreover, any integral of the kind $V^{-n}\int d^{3}R_{1}\cdots d^{3}R_{n}\;\left(\cdots \right)$ is hereafter denoted as ∫(⋯). For example, Equations (3) and (4) indicate that $S_{N}^{\mathrm{exc}}/k_{B}=-\int P_{12\ldots N}\ln P_{12\ldots N}$.

To build up the CE expansion term by term, our strategy is to consider a progressively larger number of particles. For a one-particle system, the excess entropy in units of the Boltzmann constant is $S_{1}^{\mathrm{exc}}/k_{B}=-\int P_{1}\ln P_{1}$, leading to a first-order approximation to the excess entropy of a *N*-particle system in the form $S_{N}^{\mathrm{exc}}/k_{B}\approx S_{N}^{\left(1\right)}/k_{B}\equiv -N\int P_{1}\ln P_{1}$ (that is, each particle contributes to the entropy independently of the other particles). For a two-particle system, the excess entropy is $S_{2}^{\left(1\right)}$ plus a remainder $k_{B}R_{2}$, given by:

(9) $$R_{2}\equiv \frac{S_{2}^{\mathrm{exc}}-S_{2}^{\left(1\right)}}{k_{B}}=-\int P_{12}\ln P_{12}+2\int P_{1}\ln P_{1}=-\int P_{12}\ln Q_{12}\;.$$

Equation (9) suggests a second-order approximation for $S_{N}^{\mathrm{exc}}$, where each distinct pair of particles contributes the same two-body residual term to the entropy:

(10) SN(2)kB=−N∫P1lnP1−N2∫P12lnQ12.

Notice that Equation (10) is exact for N=2, i.e., $S_{2}^{\left(2\right)}=S_{2}^{\mathrm{exc}}$. Similarly, for a three-particle system the excess entropy is $S_{3}^{\left(2\right)}$ plus a remainder $k_{B}R_{3}$:

(11) R3≡S3exc−S3(2)kB=−∫P123lnP123+3∫P1lnP1+32∫P12lnQ12=−∫P123lnQ123+32∫P12lnQ12.

Hence, a third-order approximation follows for $S_{N}^{\mathrm{exc}}$ in the form

(12) SN(3)kB=−N∫P1lnP1−N2∫P12lnQ12−N3∫P123lnQ123−32∫P12lnQ12.

Again, $S_{3}^{\left(3\right)}=S_{3}^{\mathrm{exc}}$. Equation (12) reproduces the first three terms in the rhs of Equation (5.9) of reference [8], and one may legitimately expect that the further terms in the entropy expansion are similarly obtained by arguing for N=4,5,… like we did for N=1,2,3 (see the proof in [8]).

The general entropy formula finally reads:

(13) SNexckB=−∫P12…NlnP12…N=−N∫P1lnP1−∫P12…NlnQ12…N=−N∫P1lnP1−∑n=2NNn∑a=2n(−1)n−ana∫P1…alnQ1…a.

This equation is trivially correct since, for any finite sequence $\left\{c_{a}\right\}$ of numbers,

(14) cN=∑n=2NNn∑a=2n(−1)n−anaca.

To prove (14), it is sufficient to observe that, for each fixed k=2,…,N, the coefficient of $c_{k}$ in the above sum is

(15) ∑n=kN(−1)n−kNnnk=∑n=0N−k(−1)nNn+kn+kk=Nk∑n=0N−k(−1)nN−kn=0,for2≤k<N1,fork=N.

A more compact entropy formula is

(16) SNexckB=−∑n=1NNn∑a=1n(−1)n−ana∫P1…alnP1…a,

which follows from

(17) cN=∑n=1NNn∑a=1n(−1)n−anaca.

The entropy expansion, (13) or (16), is only valid in the CE. Eliminating $Q_{1\ldots a}$ in favor of $g_{1\ldots a}$ by Equation (7), an overall constant comes out of the integral in Equation (13), namely,

(18) ∑n=2NNn∑a=2n(−1)n−analn(N−1)⋯(N−a+1)Na−1,

which, by Equation (15), equals $\ln\left(N!/N^{N}\right)$; this term exactly cancels an identical term present in the ideal-gas entropy. In the end, a modified entropy MPCE emerges:

(19) SNkB=N32−ln(ρΛ3)−N∫P1lnP1−∑n=2NNn∑a=2n(−1)n−ana∫P1…alng1…a.

Notice that the first term in the rhs differs by *N* from the ideal-gas entropy expression in the thermodynamic limit. In order that Equation (19) conforms to the GCE entropy expansion, for each *n* a suitable fluctuation integral of value −N/[n(n−1)] should be summed to (and subtracted from) the *n*-th term in the expansion. For example, using Equations (7) and (8) the second-order term in (13) can be rewritten as

(20) −N2∫d3r1d3r2V2P12lnP12P1P2=N2lnN−1N−N2∫d3r1d3r2V2P1P2g12lng12=N2lnN−1N+N2−12ρ2∫d3r1d3r2P1P2g12lng12−g12+1.

Overall, the extra constants appearing in each term of the entropy formula (for example, the quantity N/2 in Equation (20)) add to *N*. By absorbing such a *N* in the first term of (19) we recover the ideal-gas entropy in the thermodynamic limit, and the CE expansion becomes formally identical to the grand-canonical MPCE [9].

In Appendix A we present another derivation of the entropy formula in the CE, which is closer in spirit to the one given by H. S. Green. In parallel, we show that the approximation obtained by truncating the MPCE at a given order can be derived from a modified $P_{12\ldots N}$ distribution, which is an explicit functional of the spatial correlation functions up to that order.

## 3. The First Few Terms in the Expansion of Crystal Entropy

The entropy expansion in the CE is formally identical for a fluid system and a crystal, since the origin of (13) is purely combinatorial. However, the DFs of the two phases are radically different: most notably, while $P_{1}=1$ and $\rho^{\left(1\right)}=\rho$ for a homogeneous fluid, the one-body density is spatially structured for a crystal—at least once the degeneracy due to translations and point-group operations has been lifted; we stress that $P_{1}\neq 1$ only provided that a specific determination of the crystal is taken, since otherwise $P_{1}=1$ also in the “delocalized” crystalline phase. In practice, in order to fix a crystal in space we should imagine to apply a suitable symmetry-breaking external potential, whose strength is sent to zero after statistical averages have been carried out (in line with Bogoliubov’s advice to interpret statistical averages of broken-symmetry phases as quasiaverages [28], which amounts to sending the strength of the symmetry-breaking potential to zero only after the thermodynamic limit has been taken). A way to accomplish this task is to constrain the position of just one particle. When periodic boundary conditions are applied, keeping one particle fixed will be enough to break the continuous symmetries of free space. As *N* grows, the effect of the external potential becomes weaker and weaker, since it does not scale with the size of the system.

### 3.1. One-Body Entropy

A reasonable form of one-body density for a three-dimensional Bravais crystal without defects is the Tarazona ansatz [29]:

(21) $$\rho^{\left(1\right)}\left(r\right)={\left(\frac{\alpha }{\pi }\right)}^{3/2}\sum_{R}e^{-\alpha {\left(r-R\right)}^{2}}=\rho \sum_{G}e^{-\frac{G^{2}}{4\alpha }}e^{iG\cdot r}\;,$$

where α>0 is a temperature-dependent parameter, the **R**’s are direct-lattice vectors, and the **G**’s are reciprocal-lattice vectors (recall that G·R=2πm with m∈Z and $\int_{V}d^{3}r\;\exp\left\{i\left(G+G^{\prime }\right)\cdot r\right\}=V\delta_{G^{\prime },-G}$). Equation (21) is a rather generic form of crystal density, which recently we have also applied in a different context [30]. More generally, the one-body density appropriate to a perfect crystal must obey $\rho^{\left(1\right)}\left(r+R\right)=\rho^{\left(1\right)}\left(r\right)$ for all R, and is thus necessarily of the form

(22) $$\rho^{\left(1\right)}\left(r\right)=\sum_{G}{\tilde{u}}_{G}e^{iG\cdot r}\;\;\;\;\;\;\mathrm{with}\;\;\;\;\;\;{\tilde{u}}_{G}^{*}={\tilde{u}}_{-G}\;.$$

Since $\int_{V}d^{3}r\;\rho^{\left(1\right)}\left(r\right)=N$, it soon follows ${\tilde{u}}_{0}=\rho$. Calling C a primitive cell and $v_{0}$ its volume, ${\tilde{u}}_{G}=v_{0}^{-1}\int_{C}d^{3}r\;\rho^{\left(1\right)}\left(r\right)\exp\left\{-iG\cdot r\right\}\to 0$ as G→∞ (by the Riemann–Lebesgue lemma). In real space, a legitimate $\rho^{\left(1\right)}\left(r\right)$ function is $\sum_{R}\phi \left(r-R\right)$ with $\int d^{3}r\;\phi \left(r\right)=1$ (integration bounds are left unspecified when the integral is over a macroscopic *V*). In the zero-temperature/infinite-density limit, particles sit at the lattice sites and the one-body density then becomes

(23) $$\rho^{\left(1\right)}\left(r\right)=\sum_{R}\delta^{3}\left(r-R\right)\;.$$

Equation (23) is also recovered from Equation (21) in the α→∞ limit.

For a crystalline solid, the one-body entropy, that is, the first term in the expansion of excess entropy, is (in units of $k_{B}$):

(24) $$S_{1}\equiv -\frac{N}{V}\int d^{3}r_{1}\;P_{1}\ln P_{1}=-\int d^{3}r_{1}\;\rho^{\left(1\right)}\left(r_{1}\right)\ln\frac{\rho^{\left(1\right)}\left(r_{1}\right)}{\rho }\;.$$

One may wonder whether the integral in (24) is O(N) in the infinite-size limit. The answer is affirmative, and a simple argument goes as follows. Let $\rho^{\left(1\right)}\left(r\right)$ be $\sum_{R}\phi \left(r-R\right)$; if ϕ(r) is strongly localized near r=0, then $\rho^{\left(1\right)}\left(r\right)≃\phi \left(r\right)$ in the cell around R=0 and $S_{1}≃-N\int_{C}d^{3}r\;\phi \left(r\right)\ln\left\{\phi (r)/\rho \right\}=O\left(N\right)$. Actually, we can provide a rigorous proof that $S_{1}$ is negative-semidefinite and its absolute value does not grow faster than *N*. Using lnx≤x−1 for x>0 and xlnx≥x−1 for any x≥0, we obtain

(25) $$0=\rho \int d^{3}r_{1}\left(\frac{\rho_{1}}{\rho }-1\right)\leq \int d^{3}r_{1}\;\rho_{1}\ln\frac{\rho_{1}}{\rho }\leq \int d^{3}r_{1}\;\rho_{1}\left(\frac{\rho_{1}}{\rho }-1\right)=\rho^{-1}\int d^{3}r_{1}\;\rho_{1}^{2}-N\;.$$

To estimate $\int d^{3}r_{1}\;\rho_{1}^{2}$ we employ the one-body density in (21), which is sufficiently generic for our purposes:

(26) $$\rho^{-1}\int d^{3}r{\left(\rho^{\left(1\right)}\left(r\right)\right)}^{2}=\rho \sum_{G,G^{\prime }}e^{-\left(\frac{G^{2}}{4\alpha }+\frac{G^{\prime 2}}{4\alpha }\right)}\int d^{3}r\;e^{i(G+G^{\prime })\cdot r}=N\sum_{G}e^{-\frac{G^{2}}{2\alpha }}$$

(the above result is nothing but Parseval’s theorem as applied to (21)). The sum in the rhs of Equation (26) is the three-dimensional analog of a Jacobi theta function (see, e.g., [31]), whose value is O(1) for α>0. Therefore, it follows from Equations (25) and (26) that the one-body entropy is at most O(N).

### 3.2. Two-Body Entropy

We now move to the problem of evaluating the two-body entropy $S_{2}$ for a crystal. For a homogeneous fluid, $S_{2}$ is an extensive quantity which, in $k_{B}$ units, is equal to

(27) $$\mathrm{fluid}:\;S_{2}=-2\pi \rho N\int_{0}^{\infty }dr\;r^{2}\left(g(r)\ln g(r)-g(r)+1\right)\;.$$

For a crystal, we have from Equation (20) that

(28) $$S_{2}=-\frac{1}{2}\rho^{2}\int d^{3}r_{1}d^{3}r_{2}\;P_{1}P_{2}\left(g_{12}\ln g_{12}-g_{12}+1\right)\;.$$

As xlnx≥x−1 for x>0, $S_{2}$ is usually negative and zero exclusively for $g_{12}=1$. In terms of density functions, $S_{2}$ is written as

(29) $$S_{2}=-\frac{1}{2}\int d^{3}r_{1}d^{3}r_{2}\left(\rho^{\left(2\right)}\left(r_{1},r_{2}\right)\ln\frac{\rho^{\left(2\right)}\left(r_{1},r_{2}\right)}{\rho^{\left(1\right)}\left(r_{1}\right)\rho^{\left(1\right)}\left(r_{2}\right)}-\rho^{\left(2\right)}\left(r_{1},r_{2}\right)+\rho^{\left(1\right)}\left(r_{1}\right)\rho^{\left(1\right)}\left(r_{2}\right)\right)\;.$$

We show below that Equation (29) can be expressed as a radial integral, i.e., in a way similar to the two-body entropy for a fluid.

We can assign a radial structure to crystals by appealing to a couple of functions introduced in [32], namely

(30) $$\rho^{2}\tilde{g}\left(r\right)=\int \frac{d^{3}r_{1}}{V}\int \frac{d^{2}\Omega }{4\pi }\;\rho^{\left(2\right)}\left(r_{1},r_{1}+r\right)$$

and

(31) $$\rho^{2}{\tilde{g}}_{0}\left(r\right)=\int \frac{d^{3}r_{1}}{V}\int \frac{d^{2}\Omega }{4\pi }\;\rho^{\left(1\right)}\left(r_{1}\right)\rho^{\left(1\right)}\left(r_{1}+r\right)\;,$$

where the inner integrals are over the direction of **r**. For a homogeneous fluid, $\tilde{g}\left(r\right)=g\left(r\right)$ and ${\tilde{g}}_{0}\left(r\right)=1$. The authors of reference [32] have sketched the profile of $\tilde{g}\left(r\right)$ and ${\tilde{g}}_{0}\left(r\right)$ for a crystal; both functions show narrow peaks at neighbor positions in the lattice, with an extra peak at zero distance for ${\tilde{g}}_{0}\left(r\right)$, and the oscillations persist till large distances. The following sum rules hold (cf. Equation (8) for n=1):

(32) $$4\pi \int dr\;r^{2}\rho \tilde{g}\left(r\right)=\frac{1}{\rho }4\pi \int \frac{d^{3}r_{1}}{V}\rho^{\left(1\right)}\left(r_{1}\right)\underset{\frac{N-1}{4\pi }}{\underbrace{\int \frac{d^{3}r_{2}}{4\pi }\frac{\rho^{\left(2\right)}\left(r_{1},r_{2}\right)}{\rho^{\left(1\right)}\left(r_{1}\right)}}}=N-1$$

and

(33) $$4\pi \int dr\;r^{2}\rho {\tilde{g}}_{0}\left(r\right)=\frac{1}{\rho }4\pi \int \frac{d^{3}r_{1}}{V}\rho^{\left(1\right)}\left(r_{1}\right)\underset{\frac{N}{4\pi }}{\underbrace{\int \frac{d^{3}r_{2}}{4\pi }\rho^{\left(1\right)}\left(r_{2}\right)}}=N\;.$$

When the latter two formulae are rewritten as

(34) $$4\pi \rho \int dr\;r^{2}\left(\tilde{g}\left(r\right)-1\right)=-1\;\;\;\;\;\;\mathrm{and}\;\;\;\;\;\;4\pi \rho \int dr\;r^{2}\left({\tilde{g}}_{0}\left(r\right)-1\right)=0\;,$$

it becomes apparent that both $\tilde{g}\left(r\right)$ and ${\tilde{g}}_{0}\left(r\right)$ decay to 1 at infinity. Similarly, we define:

(35) $$\rho^{2}\tilde{h}\left(r\right)=\int \frac{d^{3}r_{1}}{V}\int \frac{d^{2}\Omega }{4\pi }\;\rho^{\left(2\right)}\left(r_{1},r_{1}+r\right)\ln\frac{\rho^{\left(2\right)}\left(r_{1},r_{1}+r\right)}{\rho^{\left(1\right)}\left(r_{1}\right)\rho^{\left(1\right)}\left(r_{1}+r\right)}\;,$$

which obviously vanishes at infinity. While $\tilde{h}\left(r\right)=g\left(r\right)\ln g\left(r\right)$ for a homogeneous fluid, we expect that $\tilde{h}\left(r\right)\neq \tilde{g}\left(r\right)\ln\tilde{g}\left(r\right)$ in the crystal. Putting Equations (30)–(35) together, we arrive at

(36) $$\mathrm{crystal}:\;S_{2}=-2\pi \rho N\int_{0}^{\infty }dr\;r^{2}\left(\tilde{h}\left(r\right)-\tilde{g}\left(r\right)+{\tilde{g}}_{0}\left(r\right)\right)=-2\pi \rho N\int_{0}^{\infty }dr\;r^{2}\tilde{h}\left(r\right)-\frac{N}{2}\;.$$

Even though the integrand vanishes at infinity, $S_{2}=O\left(N\right)$ only if the envelope of $\tilde{h}\left(r\right)$ decays faster than $r^{-3}$ ($r^{-2}$ in two dimensions). A slower decay may be sufficient if $S_{2}$ is computed through the first integral in (36). For a spherically-symmetric interaction potential, the excess energy (i.e., the canonical average of the total potential energy *U*) can also be written as a radial integral:

(37) $$\begin{array}{ccc} \langle U\rangle & = & \frac{1}{2}\int d^{3}r_{1}d^{3}r_{2}\;\rho^{\left(2\right)}\left(r_{1},r_{2}\right)u(|r_{2}-r_{1}\left|\right)\\ & = & \frac{1}{2}\int_{0}^{\infty }dr\;r^{2}u\left(r\right)\int d^{3}r_{1}\int d^{2}\Omega \;\rho^{\left(2\right)}\left(r_{1},r_{1}+r\right)=2\pi \rho N\int_{0}^{\infty }dr\;r^{2}u\left(r\right)\tilde{g}\left(r\right)\;. \end{array}$$

For the one-body density in (21), ${\tilde{g}}_{0}\left(r\right)$ can be obtained in closed form. First we have:

(38) $$\int \frac{d^{2}\Omega }{4\pi }\;\rho^{\left(1\right)}\left(r_{1}+r\right)=\rho \sum_{G}e^{-\frac{G^{2}}{4\alpha }}e^{iG\cdot r_{1}}\frac{\sin(Gr)}{Gr}\;.$$

Then, multiplying by $\rho^{\left(1\right)}\left(r_{1}\right)=\rho \sum_{G^{\prime }}e^{-\frac{G^{\prime 2}}{4\alpha }}e^{iG^{\prime }\cdot r_{1}}$ and finally integrating over $r_{1}$ we arrive at

(39) $${\tilde{g}}_{0}\left(r\right)=1+\sum_{G\neq 0}e^{-\frac{G^{2}}{2\alpha }}\frac{\sin(Gr)}{Gr}\;.$$

We see that the large-distance decay of ${\tilde{g}}_{0}\left(r\right)$ is usually slow, and the same will occur for $\tilde{g}\left(r\right)$ since $\tilde{g}\left(r\right)≃{\tilde{g}}_{0}\left(r\right)$ for large *r*. In two dimensions, the one-body density and ${\tilde{g}}_{0}$ functions respectively read:

(40) $$\rho^{\left(1\right)}\left(r\right)=\frac{\alpha }{\pi }\sum_{R}e^{-\alpha {\left(r-R\right)}^{2}}=\rho \sum_{G}e^{-\frac{G^{2}}{4\alpha }}e^{iG\cdot r}\;\;\;\mathrm{and}\;\;\;{\tilde{g}}_{0}\left(r\right)=1+\sum_{G\neq 0}e^{-\frac{G^{2}}{2\alpha }}J_{0}\left(Gr\right)\;,$$

where $J_{0}$ is a Bessel function of the first kind. Since the envelope of $J_{0}$ maxima decays as $r^{-1/2}$ at infinity, we see that the asymptotic vanishing of ${\tilde{g}}_{0}$ is slower in two dimensions than in three.

Equation (39) has a definite limit for α→∞, corresponding to zero temperature. Indeed, using Poisson summation formula and the expression of Dirac’s delta in spherical coordinates, we obtain:

(41) $$\begin{array}{ccc} \rho {\tilde{g}}_{0}\left(r\right) & = & \rho \left(1+\sum_{G\neq 0}\frac{\sin(Gr)}{Gr}\right)=\rho \int \frac{d^{2}\Omega }{4\pi }\sum_{G}e^{iG\cdot r}=\delta^{3}\left(r\right)+\int \frac{d^{2}\Omega }{4\pi }\sum_{R\neq 0}\delta^{3}\left(r-R\right)\\ & = & \delta^{3}\left(r\right)+\sum_{R\neq 0}\frac{1}{4\pi }\int_{0}^{2\pi }d\phi \int_{0}^{\pi }d\theta \;\sin\theta \frac{1}{r^{2}\sin\theta }\delta \left(r-R\right)\delta \left(\theta -\theta_{R}\right)\delta \left(\phi -\phi_{R}\right)\\ & = & \delta^{3}\left(r\right)+\sum_{R\neq 0}\frac{1}{4\pi R^{2}}\delta \left(r-R\right)\;. \end{array}$$

Hence, ${\tilde{g}}_{0}\left(r\right)$ reduces to a sum of delta functions centered at lattice distances (including the origin). The latter result is actually general. Inserting Equation (23) in (31), we obtain:

(42) $$\begin{array}{ccc} \rho {\tilde{g}}_{0}\left(r\right) & = & \frac{1}{\rho }\int \frac{d^{3}r_{1}}{V}\int \frac{d^{2}\Omega }{4\pi }\sum_{R}\delta^{3}\left(r_{1}-R\right)\sum_{R^{\prime }}\delta^{3}\left(r_{1}+r-R^{\prime }\right)\\ & = & \frac{1}{\rho }\int \frac{d^{3}r_{1}}{V}\int \frac{d^{2}\Omega }{4\pi }\left\{\sum_{R}\delta^{3}\left(r_{1}-R\right)\delta^{3}\left(r_{1}+r-R\right)+\sum_{R\neq R^{\prime }}\delta^{3}\left(r_{1}-R\right)\delta^{3}\left(r_{1}+r-R^{\prime }\right)\right\}\\ & = & \frac{1}{\rho }\int \frac{d^{3}r_{1}}{V}\int \frac{d^{2}\Omega }{4\pi }\left\{\sum_{R}\delta^{3}\left(r_{1}-R\right)\delta^{3}\left(r\right)+\sum_{R\neq R^{\prime }}\delta^{3}\left(r_{1}-R\right)\delta^{3}\left(r_{1}+r-R^{\prime }\right)\right\}\\ & = & \frac{1}{\rho }\sum_{R}\delta^{3}\left(r\right)\int \frac{d^{3}r_{1}}{V}\delta^{3}\left(r_{1}-R\right)+\frac{1}{\rho }\sum_{R\neq R^{\prime }}\int \frac{d^{3}r_{1}}{V}\delta^{3}\left(r_{1}-R\right)\frac{\delta (r-|r_{1}-R^{\prime }|)}{4\pi |r_{1}-R^{\prime }{|}^{2}}\\ & = & \delta^{3}\left(r\right)+\sum_{R\neq 0}\frac{1}{4\pi R^{2}}\delta \left(r-R\right)\;, \end{array}$$

q.e.d. At zero temperature, $\rho \tilde{g}\left(r\right)$ is given by the same sum of delta-function terms as in (42), but for the first term, $\delta^{3}\left(r\right)$, which is missing—see Equation (92) below.

We add a final comment on possible alternative formulations of $\tilde{g}\left(r\right)$ for a crystal. One choice is to replace (30) with

(43) $$\mathrm{option}\;\;B:\;\rho \tilde{g}\left(r\right)=\int \frac{d^{3}r_{1}}{V}\int \frac{d^{2}\Omega }{4\pi }\frac{\rho^{\left(2\right)}\left(r_{1},r_{1}+r\right)}{\rho^{\left(1\right)}\left(r_{1}\right)}\;.$$

Apparently, this is a good definition since (see Equation (8))

(44) $$4\pi \int dr\;r^{2}\rho \tilde{g}\left(r\right)=4\pi \underset{1}{\underbrace{\int \frac{d^{3}r_{1}}{V}}}\underset{\frac{N-1}{4\pi }}{\underbrace{\int \frac{d^{3}r_{2}}{4\pi }\frac{\rho^{\left(2\right)}\left(r_{1},r_{2}\right)}{\rho^{\left(1\right)}\left(r_{1}\right)}}}=N-1\;.$$

However, with this $\tilde{g}\left(r\right)$ we cannot write $S_{2}$ as a radial integral—hence, option B is discarded altogether. Another possibility is

(45) $$\mathrm{option}\;\;C:\;\tilde{g}\left(r\right)=\int \frac{d^{3}r_{1}}{V}\int \frac{d^{2}\Omega }{4\pi }\frac{\rho^{\left(2\right)}\left(r_{1},r_{1}+r\right)}{\rho^{\left(1\right)}\left(r_{1}\right)\rho^{\left(1\right)}\left(r_{1}+r\right)}\;,$$

but this option is useless too, since

(46) $$4\pi \int dr\;r^{2}\rho \tilde{g}\left(r\right)=\rho \int d^{3}r_{1}\frac{1}{V}\int d^{3}r_{2}\;g^{\left(2\right)}\left(r_{1},r_{2}\right)=\;?$$

(observe that the inner integral is different from the one appearing in Equation (8)).

### 3.3. Symmetries of the Two-Body Density

A general property of the two-body density for a crystal is the CE sum rule

(47) $$\int d^{3}r_{2}\;\rho^{\left(2\right)}\left(r_{1},r_{2}\right)=\left(N-1\right)\rho^{\left(1\right)}\left(r_{1}\right)\;.$$

Other constraints follow from the translational symmetry of local crystal properties. As for the one-body density, fulfilling $\rho^{\left(1\right)}\left(r_{1}+R\right)=\rho^{\left(1\right)}\left(r_{1}\right)$ for every R, we must have that

(48) $$\rho^{\left(2\right)}\left(r_{1},r_{2}\right)=\rho^{\left(2\right)}\left(r_{1}+R,r_{2}+R\right)\;,$$

in turn implying

(49) $$g^{\left(2\right)}\left(r_{1},r_{2}\right)=g^{\left(2\right)}\left(r_{1}+R,r_{2}+R\right)\;.$$

Now observe [33] that (i) any function of $r_{1}$ and $r_{2}$ can also be viewed as a function of $(r_{1}+r_{2})/2$ and $r_{2}-r_{1}$; (ii) under a R-translation, only the former variable is affected, not the relative separation. Hence, the most general function consistent with (49) is:

(50) $$g^{\left(2\right)}\left(r_{1},r_{2}\right)=\sum_{G}{\tilde{v}}_{G}\left(r_{2}-r_{1}\right)e^{iG\cdot \frac{r_{1}+r_{2}}{2}}\;,$$

where

(51) $$g^{\left(2\right)}\left(r_{1},r_{2}\right)\in R⟹{\tilde{v}}_{G}^{*}\left(r_{2}-r_{1}\right)={\tilde{v}}_{-G}\left(r_{2}-r_{1}\right)$$

and

(52) $$g^{\left(2\right)}\left(r_{1},r_{2}\right)=g^{\left(2\right)}\left(r_{2},r_{1}\right)⟹{\tilde{v}}_{G}\left(r_{2}-r_{1}\right)={\tilde{v}}_{G}\left(r_{1}-r_{2}\right)\;.$$

In order that $\lim_{r\to \infty }g^{\left(2\right)}\left(r_{1},r_{1}+r\right)=1$ it is sufficient that

(53) $$\lim_{r\to \infty }{\tilde{v}}_{0}\left(r\right)=1\;\;\;\;\;\;\mathrm{and}\;\;\;\;\;\;\lim_{r\to \infty }{\tilde{v}}_{G}\left(r\right)=0\;\;\mathrm{for}\;\;G\neq 0\;.$$

We may reasonably expect that the most relevant term in the expansion (50) is indeed the G=0 one (also notice that ${\tilde{v}}_{G}\to 0$ as G→∞ by the Riemann–Lebesgue lemma).

Equation (50) is still insufficient to establish the scaling of two-body entropy with the size of the crystal. Some general results can be obtained under the (strong) assumption that ${\tilde{v}}_{G}\left(r\right)=0$ for any G≠0. If we change the notation from ${\tilde{v}}_{0}$ to G(r)≡1+H(r) (which, by Equations (51) and (52), is a real and even function), then a necessary condition for H is:

(54) $$\int d^{3}r_{2}\;\rho^{\left(1\right)}\left(r_{2}\right)H\left(r_{2}-r_{1}\right)=-1\;\;\;\;\;\;\mathrm{for}\;\;\mathrm{any}\;\;r_{1}\;\;\mathrm{where}\;\;\rho^{\left(1\right)}\left(r_{1}\right)\neq 0\;.$$

The rationale behind Equation (54) is particularly transparent near T=0, where the peaks of the one-body density are extremely narrow. As argued below (see Equation (67) ff.), H as a function of $r_{2}$ is roughly −1 in the primitive cell C centered in $r_{1}\approx R_{1}$, $R_{1}$ denoting the only lattice site contained in C and roughly zero outside C. Since the integral of $\rho^{\left(1\right)}$ over C equals 1, Equation (54) will immediately follow.

Now writing H(r) as a Fourier integral,

(55) $$H\left(r\right)=\int \frac{d^{3}k}{{\left(2\pi \right)}^{3}}\tilde{H}\left(k\right)e^{ik\cdot r}\;,$$

and using (21) as one-body density, Equation (54) yields

(56) $$\rho \sum_{G}e^{-\frac{G^{2}}{4\alpha }}\tilde{H}\left(G\right)e^{-iG\cdot r_{1}}=-1\;,$$

which can only hold for arbitrary $r_{1}$ if

(57) $$\tilde{H}\left(G\right)=-\frac{1}{\rho }\delta_{G,0}\;.$$

Next, from Equation (30) we obtain:

(58) $$\rho^{2}\tilde{g}\left(r\right)=\rho^{2}{\tilde{g}}_{0}\left(r\right)+\int \frac{d^{3}r_{1}}{V}\rho^{\left(1\right)}\left(r_{1}\right)\int \frac{d^{2}\Omega }{4\pi }\;\rho^{\left(1\right)}\left(r_{1}+r\right)H\left(r\right)\;.$$

For the one-body density in (21), the inner integral becomes:

(59) $$\int \frac{d^{2}\Omega }{4\pi }\;\rho^{\left(1\right)}\left(r_{1}+r\right)H\left(r\right)=\rho \sum_{G}e^{-\frac{G^{2}}{4\alpha }}I_{G}\left(r\right)e^{-iG\cdot r_{1}}$$

with

(60) $$I_{G}\left(r\right)=\int \frac{d^{2}\Omega }{4\pi }\;H\left(r\right)e^{-iG\cdot r}=\int \frac{d^{3}k}{{\left(2\pi \right)}^{3}}\;\tilde{H}\left(k\right)\frac{\sin\left(|k-G|r\right)}{|k-G|r}\;.$$

It is evident that $I_{G}\left(r\right)$ vanishes at infinity. Upon inserting (59) in (58), we finally obtain:

(61) $$\tilde{g}\left(r\right)={\tilde{g}}_{0}\left(r\right)+\sum_{G}e^{-\frac{G^{2}}{2\alpha }}I_{G}\left(r\right)\;.$$

As *r* increases, the second term gradually vanishes and the large-distance oscillations of $\tilde{g}\left(r\right)$ then exactly match those of ${\tilde{g}}_{0}\left(r\right)$. As a countercheck, let us compute the integral of $\rho \tilde{g}\left(r\right)-\rho {\tilde{g}}_{0}\left(r\right)$ over the macroscopic system volume (which, by Equations (32) and (33), should be −1):

(62) $$\begin{array}{ccc} 4\pi \int dr\;r^{2}\rho \left(\tilde{g}\left(r\right)-{\tilde{g}}_{0}\left(r\right)\right) & = & \rho \sum_{G}e^{-\frac{G^{2}}{2\alpha }}\cdot 4\pi \int dr\;r^{2}I_{G}\left(r\right)\\ & = & \rho \sum_{G}e^{-\frac{G^{2}}{2\alpha }}\int \frac{d^{3}k}{{\left(2\pi \right)}^{3}}\tilde{H}\left(k\right)\cdot 4\pi \int dr\;r^{2}\frac{\sin\left(|k-G|r\right)}{|k-G|r}\\ & = & \rho \sum_{G}e^{-\frac{G^{2}}{2\alpha }}\int \frac{d^{3}k}{{\left(2\pi \right)}^{3}}\tilde{H}\left(k\right)\underset{{\left(2\pi \right)}^{3}\delta^{3}\left(k-G\right)}{\underbrace{\int d^{3}r\;e^{i(k-G)\cdot r}}}\\ & = & \rho \sum_{G}e^{-\frac{G^{2}}{2\alpha }}\underset{-\left(1/\rho \right)\delta_{G,0}}{\underbrace{\tilde{H}\left(G\right)}}=-1\;. \end{array}$$

Under the assumption that

(63) $$\rho^{\left(2\right)}\left(r_{1},r_{1}+r\right)=\rho^{\left(1\right)}\left(r_{1}\right)\rho^{\left(1\right)}\left(r_{1}+r\right)G\left(r\right)\;,$$

the entropy expansion for a crystal reads

(64) $$\begin{array}{ccc} \frac{S}{k_{B}}= & & -\int d^{3}r_{1}\;\rho^{\left(1\right)}\left(r_{1}\right)\ln\frac{\rho^{\left(1\right)}\left(r_{1}\right)}{\rho }\\ & & -\frac{1}{2}\int d^{3}r_{1}\;\rho^{\left(1\right)}\left(r_{1}\right)\int d^{3}r\;\rho^{\left(1\right)}\left(r_{1}+r\right)\left[G(r)\ln G(r)-G(r)+1\right]+\ldots \end{array}$$

Providing that it vanishes sufficiently rapidly at infinity, the function

(65) K(r)=G(r)lnG(r)−G(r)+1

can be written as a Fourier integral, and using (21) as one-body density, the two-body entropy becomes

(66) $$S_{2}=-\frac{1}{2}\rho^{2}\sum_{G,G^{\prime }}e^{-\frac{G^{2}+G^{\prime 2}}{4\alpha }}\underset{V\delta_{G^{\prime },-G}}{\underbrace{\int d^{3}r_{1}\;e^{i\left(G+G^{\prime }\right)\cdot r_{1}}}}\int d^{3}r\;K\left(r\right)e^{iG^{\prime }\cdot r}=-\frac{1}{2}N\rho \sum_{G}e^{-\frac{G^{2}}{2\alpha }}\tilde{K}\left(G\right)\;,$$

which is clearly O(N).

### 3.4. Two-Body Density at T=0

In the zero-temperature limit, particles will be sitting at lattice sites, and the two-body density then becomes (see Equation (23)):

(67) $$\rho^{\left(2\right)}\left(r_{1},r_{2}\right)=\underset{R,R^{\prime }}{\sum^{\prime }}\delta^{3}\left(r_{1}-R\right)\delta^{3}\left(r_{2}-R^{\prime }\right)=\rho^{\left(1\right)}\left(r_{1}\right)\rho^{\left(1\right)}\left(r_{2}\right)\left(1-1_{C}\left(r_{2}-r_{1}\right)\right)\;,$$

which is of the form (63). In Equation (67), $1_{C}\left(r\right)$ is the indicator function of a Wigner–Seitz cell C centered at the origin (i.e., $1_{C}\left(r\right)=1$ if r∈C and $1_{C}\left(r\right)=0$ otherwise). While the factor $\rho^{\left(1\right)}\left(r_{1}\right)\rho^{\left(1\right)}\left(r_{2}\right)$ forces particles to be located at lattice sites, the only role of the G in (67) is to prevent the possibility of double site occupancy. However, a G function with this property is not unique; the one provided in (67) has the advantage of exactly complying with condition (57) (see below). Equation (67) indicates that the pair-correlation structure of a low-temperature solid is very different from the structure of a dense fluid close to freezing.

For

(68) $$H\left(r\right)=-1_{C}\left(r\right)=\left\{\begin{array}{cc} -1, & \;\;\;\mathrm{for}\;\;r\in C\\ 0, & \;\;\;\mathrm{otherwise} \end{array}\right.$$

the Fourier transform reads:

(69) $$\begin{array}{ccc} \tilde{H}\left(k\right) & = & \int d^{3}r\;H\left(r\right)e^{-ik\cdot r}=-\int_{C}d^{3}r\;e^{-ik\cdot r}\;. \end{array}$$

Now observe that f(r)=1 is trivially periodic, and can thus be expanded in plane waves as $1=\sum_{G}{\tilde{f}}_{G}e^{iG\cdot r}$, with ${\tilde{f}}_{G}=\delta_{G,0}$. On the other hand,

(70) $${\tilde{f}}_{G}=\frac{1}{v_{0}}\int_{C}d^{3}r\;f\left(r\right)e^{-iG\cdot r}=\rho \int_{C}d^{3}r\;e^{-iG\cdot r}\;.$$

Comparing Equations (69) and (70), we conclude that

(71) $$\tilde{H}\left(G\right)=-\frac{1}{\rho }\delta_{G,0}\;.$$

For $H\left(r\right)=-1_{C}\left(r\right)$ the function $I_{G}\left(r\right)$ at Equation (60) equals −sin(Gr)/(Gr) for $r<r_{m}$ and 0 for $r>r_{M}$, where $r_{m}$ ($r_{M}$) is the radius of the largest (smallest) sphere inscribed in (circumscribed to) C. It then follows from Equation (61) that $\tilde{g}\left(r\right)=0$ for $r<r_{m}$, while $\tilde{g}\left(r\right)={\tilde{g}}_{0}\left(r\right)$ for $r>r_{M}$ (for a triangular crystal with spacing *a* we have $r_{m}=a/2$ and $r_{M}=a/\sqrt{3}$, both comprised between the first, 0, and the second, *a*, lattice distance). T=0, where ${\tilde{g}}_{0}\left(r\right)$ consists of infinitely narrow peaks centered at lattice distances; this implies that $\tilde{g}\left(r\right)={\tilde{g}}_{0}\left(r\right)$ everywhere but at the origin, where $\tilde{g}\left(r\right)=0$ while ${\tilde{g}}_{0}\left(r\right)$ is non-zero.

### 3.5. Scaling of Two-Body Entropy with *N*

We henceforth discuss in fully general terms how the two-body entropy scales with *N* for a crystal, avoiding to make any simplifying hypothesis on the structure of $g^{\left(2\right)}\left(r_{1},r_{2}\right)$. Using an obvious short-hand notation, the two-body entropy reads

(72) $$S_{2}=-\frac{1}{2}\int d1\;d2\left(\rho_{12}\ln\frac{\rho_{12}}{\rho_{1}\rho_{2}}-\rho_{12}+\rho_{1}\rho_{2}\right)=-\frac{1}{2}\int d1\;d2\;\rho_{1}\rho_{2}\left(g_{12}\ln g_{12}-g_{12}+1\right)\;.$$

As we already know, $S_{2}\leq 0$. From the inequality lnx≤x−1, valid for all x>0, we derive $-x\ln x\geq x-x^{2}$ for x≥0, and then obtain:

(73) $$S_{2}=\frac{1}{2}\int d1\;d2\;\rho_{1}\rho_{2}\left(-g_{12}\ln g_{12}+g_{12}-1\right)\geq -\frac{1}{2}\int d1\;d2\;\rho_{1}\rho_{2}{\left(g_{12}-1\right)}^{2}\;.$$

Clearly, estimating the size of the lower bound in Equation (73) is a much simpler problem than working with $S_{2}$ itself.

Taking $h_{12}\equiv g_{12}-1$, it is evident that $h_{12}$ shares all symmetries of $g_{12}$. Hence, we can write:

(74) $$h_{12}=\sum_{G}{\tilde{h}}_{G}\left(r_{1}-r_{2}\right)e^{iG\cdot \frac{r_{1}+r_{2}}{2}}\;\;\;\;\;\;\mathrm{with}\;\;\;\;\;\;{\tilde{h}}_{G}^{*}\left(r\right)={\tilde{h}}_{-G}\left(r\right)\;\;\;\mathrm{and}\;\;\;{\tilde{h}}_{G}\left(r\right)={\tilde{h}}_{G}\left(-r\right)\;.$$

Observe that the ${\tilde{h}}_{G}\left(r\right)$ functions are nothing but Fourier coefficients, once the *h* function has been expressed in terms of $S=(r_{1}+r_{2})/2$ and $r=r_{1}-r_{2}$:

(75) $${\tilde{h}}_{G}\left(r\right)=\frac{1}{v_{0}}\int_{C}d^{3}S\;h\left(S+r/2,S-r/2\right)e^{-iG\cdot S}\;.$$

By the Riemann–Lebesgue lemma, ${\tilde{h}}_{G}\left(r\right)\to 0$ as G→∞ (for arbitrary r). Moreover, ${\tilde{h}}_{G}\left(r\right)\to 0$ for r→∞ (for arbitrary G) since $\rho_{12}\to \rho_{1}\rho_{2}$ for $|r_{1}-r_{2}|\to \infty$. Similarly, for $k_{12}\equiv h_{12}^{2}$ we have that

(76) $$k_{12}=\sum_{G}{\tilde{k}}_{G}\left(r_{1}-r_{2}\right)e^{iG\cdot \frac{r_{1}+r_{2}}{2}}\;\;\;\;\;\;\mathrm{with}\;\;\;\;\;\;{\tilde{k}}_{G}\left(r\right)=\sum_{G^{\prime }}{\tilde{h}}_{G-G^{\prime }}\left(r\right){\tilde{h}}_{G^{\prime }}\left(r\right)\;.$$

Now observe that, for $\rho^{\left(1\right)}\left(r\right)=\sum_{G}{\tilde{u}}_{G}e^{iG\cdot r}$,

(77) $$\rho^{\left(1\right)}\left(r\right)\rho^{\left(1\right)}\left(r^{\prime }\right)=\sum_{G}\underset{{\tilde{v}}_{G}^{\infty }\left(r-r^{\prime }\right)}{\underbrace{\left(\sum_{G^{\prime }}{\tilde{u}}_{G-G^{\prime }}{\tilde{u}}_{G^{\prime }}e^{i\left(2G^{\prime }-G\right)\cdot \frac{r-r^{\prime }}{2}}\right)}}e^{iG\cdot \frac{r+r^{\prime }}{2}}\;.$$

Using the above equation, and changing the integration variables from $r_{1}$ and $r_{2}$ to S and r, we obtain:

(78) $$\begin{array}{ccc} -\frac{1}{2}\int d1\;d2\;\rho_{1}\rho_{2}{\left(g_{12}-1\right)}^{2} & = & -\frac{1}{2}\int d^{3}S\;d^{3}r\sum_{G}{\tilde{v}}_{G}^{\infty }\left(r\right)e^{iG\cdot S}\sum_{G^{\prime }}{\tilde{k}}_{G^{\prime }}\left(r\right)e^{iG^{\prime }\cdot S}\\ & = & -\frac{1}{2}\sum_{G,G^{\prime }}\underset{V\delta_{G^{\prime },-G}}{\underbrace{\int d^{3}S\;e^{i(G+G^{\prime })\cdot S}}}\underset{i_{G,G^{\prime }}}{\underbrace{\int d^{3}r\;{\tilde{v}}_{G}^{\infty }\left(r\right){\tilde{k}}_{G^{\prime }}\left(r\right)}}\\ & = & -\frac{1}{2}V\sum_{G}i_{G,-G}\;, \end{array}$$

where

(79) $$i_{G,-G}=\sum_{G^{\prime }}{\tilde{u}}_{G-G^{\prime }}{\tilde{u}}_{G^{\prime }}\int d^{3}r\left(\sum_{G^{\prime \prime }}{\tilde{h}}_{-G-G^{\prime \prime }}\left(r\right){\tilde{h}}_{G^{\prime \prime }}\left(r\right)\right)e^{i\left(2G^{\prime }-G\right)\cdot \frac{r}{2}}\;.$$

In the special case ${\tilde{h}}_{G}=H\left(r\right)\delta_{G,0}$, we have $h_{12}={\tilde{h}}_{0}=H\left(r_{1}-r_{2}\right)$ and ${\tilde{k}}_{G}\left(r\right)=H{\left(r\right)}^{2}\delta_{G,0}$. Then, from Equation (77) we derive

(80) $$\sum_{G}i_{G,-G}=i_{0,0}=\int d^{3}r\;{\tilde{v}}_{0}^{\infty }\left(r\right){\tilde{k}}_{0}\left(r\right)=\sum_{G}{\left|{\tilde{u}}_{G}\right|}^{2}\tilde{H^{2}}\left(G\right)\;.$$

An independent computation of the integral leads to the same result:

(81) $$\begin{array}{ccc} -\frac{1}{2}\int d1\;d2\;\rho_{1}\rho_{2}{\left(g_{12}-1\right)}^{2} & = & -\frac{1}{2}\int d^{3}r\;\rho^{\left(1\right)}\left(r\right)\int d^{3}r^{\prime }\;\underset{\sum_{G}{\tilde{u}}_{G}^{*}e^{-iG\cdot \left(r+r^{\prime }\right)}}{\underbrace{\rho^{\left(1\right)}\left(r+r^{\prime }\right)}}H^{2}\left(r^{\prime }\right)\\ & = & -\frac{1}{2}\sum_{G}{\tilde{u}}_{G}^{*}\underset{V{\tilde{u}}_{G}}{\underbrace{\int d^{3}r\;\rho^{\left(1\right)}\left(r\right)e^{-iG\cdot r}}}\underset{\tilde{H^{2}}\left(G\right)}{\underbrace{\int d^{3}r^{\prime }\;H^{2}\left(r^{\prime }\right)e^{-iG\cdot r^{\prime }}}}\\ & = & -\frac{1}{2}V\sum_{G}{\left|{\tilde{u}}_{G}\right|}^{2}\tilde{H^{2}}\left(G\right)\;, \end{array}$$

which should be compared with Equation (66). For $H\left(r\right)=-1_{C}\left(r\right)$ and ${\tilde{u}}_{G}=\rho \exp\left\{-G^{2}/\left(4\alpha \right)\right\}$, we readily obtain $S_{2}=-N/2$ from both Equations (66) and (78), meaning that in this case the two-body entropy coincides with its lower bound in Equation (73).

The quantity (80) is clearly O(1), since the summand is rapidly converging to zero; this implies that the two-body entropy of a crystal is, at least for ${\tilde{h}}_{G}=H\left(r\right)\delta_{G,0}$, bounded from below by a O(N) quantity. In the most general case, where Equations (78) and (79), rather, apply, we can only observe the following. As *G* grows in size, for any fixed $G^{\prime }$ and $G^{\prime \prime }$ both ${\tilde{u}}_{G-G^{\prime }}{\tilde{u}}_{G^{\prime }}$ and ${\tilde{h}}_{-G-G^{\prime \prime }}\left(r\right){\tilde{h}}_{G^{\prime \prime }}\left(r\right)$ get smaller, suggesting that $i_{G,-G}$ will decrease too. However, this is not enough to conclude that $\sum_{G}i_{G,-G}$ is O(1), and the only way to settle the problem is numerical.

### 3.6. Numerical Evaluation of the Structure Functions

The utility of (36) clearly relies on the possibility of determining the integrand in simulation with sufficient accuracy. First we see how the one-body entropy, Equation (24), is computed. We start dividing *V* into a large number $M=V/v_{c}$ of identical cubes of volume $v_{c}$, chosen to be small enough that a cube contains the center of at most one particle. Let $c_{\alpha }=0,1$ (with α=1,…,M) be the occupancy of the αth cube in a given system configuration and $\langle c_{\alpha }\rangle$ its canonical average as computed in a long Monte Carlo simulation of the weakly constrained crystal (to fix the center of mass of the crystal in space it is sufficient to keep one particle fixed; then, periodic boundary conditions will contribute to keep crystalline axes also fixed in the course of simulation). Given this setup, the local density at $r_{1}$ (a point inside the αth cube) can be estimated as

(82) $$\rho^{\left(1\right)}\left(r_{1}\right)\approx \frac{\langle c_{\alpha }\rangle }{v_{c}}\;,$$

and the integral in (24) becomes

(83) $$\int d^{3}r_{1}\;\rho^{\left(1\right)}\left(r_{1}\right)\ln\frac{\rho^{\left(1\right)}\left(r_{1}\right)}{\rho }\approx \sum_{\alpha =1}^{M}\langle c_{\alpha }\rangle \ln\frac{\langle c_{\alpha }\rangle }{\rho v_{c}}$$

(notice that $\rho v_{c}=N/M\ll 1$; we need $v_{c}\to 0$ and an infinitely long simulation to make (82) an exact relation). Similarly, if $r_{2}$ falls within the βth cube, then

(84) $$\rho^{\left(2\right)}\left(r_{1},r_{2}\right)\approx \frac{\langle c_{\alpha }c_{\beta }\rangle }{v_{c}^{2}}$$

and from Equation (30) we derive

(85) $$\begin{array}{ccc} \tilde{g}\left(r\right) & \approx & \frac{1}{\rho^{2}V}\sum_{\alpha =1}^{M}v_{c}\frac{1}{N_{\gamma }}\sum_{|\gamma |=r}\frac{\langle c_{\alpha }c_{\alpha +\gamma }\rangle }{v_{c}^{2}}=\frac{1}{\rho^{2}V}\langle \sum_{\alpha =1}^{M}v_{c}\frac{1}{N_{\gamma }}\sum_{|\gamma |=r}\frac{c_{\alpha }c_{\alpha +\gamma }}{v_{c}^{2}}\rangle \\ & = & \frac{1}{M\rho^{2}v_{c}^{2}}\langle \sum_{\alpha =1}^{M}\delta_{c_{\alpha },1}\frac{1}{N_{\gamma }}\sum_{|\gamma |=r}c_{\alpha +\gamma }\rangle \;. \end{array}$$

In the above formula $N_{\gamma }≃4\pi r^{2}\Delta r/v_{c}$ is the number of cubes whose center lies at a distance *r* from α (to within a certain tolerance Δr≪r), and the inner sum is carried out over those cubes only. Since $\rho v_{c}N_{\gamma }=4\pi r^{2}\Delta r\rho$ and $M\rho v_{c}=N$, an equivalent formula for $\tilde{g}\left(r\right)$ is

(86) $$\tilde{g}\left(r\right)\approx \langle \frac{1}{N}\sum_{i=1}^{N}\frac{N_{i}\left(r\pm \Delta r/2\right)}{4\pi r^{2}\Delta r\rho }\rangle \;,$$

denoting $N_{i}\left(r\pm \Delta r/2\right)$ the number of particles found at a distance between r−Δr/2 and r+Δr/2 from the *i*th particle in the given configuration. Equation (86) closely reflects the method of computing the radial distribution function in a CE simulation (see, e.g., Equation (11) in reference [34]).

The function $\tilde{g}\left(r\right)$ admits yet another expression, which further strengthens its resemblance to the g(r) of a liquid (as reported e.g., in [35]). It follows from Equations (30) and (6) that

(87) $$\rho^{2}\tilde{g}\left(r\right)=\frac{1}{V}\int \frac{d^{2}\Omega }{4\pi }\langle \underset{ij}{\sum^{\prime }}\int d^{3}r_{1}\;\delta^{3}\left(r_{1}-R_{i}\right)\delta^{3}\left(r_{1}+r-R_{j}\right)\rangle \;.$$

Note that, for any sufficiently smooth function f(r),

(88) $$\begin{array}{ccc} \int d^{3}r\;f\left(r\right)\int d^{3}r_{1}\;\delta^{3}\left(r_{1}-R_{i}\right)\delta^{3}\left(r_{1}+r-R_{j}\right) & = & \int d^{3}r_{1}\;\delta^{3}\left(r_{1}-R_{i}\right)\int d^{3}r\;f\left(r\right)\delta^{3}\left(r_{1}+r-R_{j}\right)\\ & = & \int d^{3}r_{1}\;\delta^{3}\left(r_{1}-R_{i}\right)f\left(R_{j}-r_{1}\right)=f\left(R_{j}-R_{i}\right) \end{array}$$

and

(89) $$\begin{array}{ccc} \int d^{3}r\;f\left(r\right)\int d^{3}r_{1}\;\delta^{3}\left(r_{1}-R_{i}\right)\delta^{3}\left(R_{i}+r-R_{j}\right) & = & \int d^{3}r_{1}\;\delta^{3}\left(r_{1}-R_{i}\right)\int d^{3}r\;f\left(r\right)\delta^{3}\left(R_{i}+r-R_{j}\right)\\ & = & f\left(R_{j}-R_{i}\right)\underset{1}{\underbrace{\int d^{3}r_{1}\;\delta^{3}\left(r_{1}-R_{i}\right)}}=f\left(R_{j}-R_{i}\right)\;, \end{array}$$

we are allowed to replace $\delta^{3}\left(r_{1}-R_{i}\right)\delta^{3}\left(r_{1}+r-R_{j}\right)$ with $\delta^{3}\left(r_{1}-R_{i}\right)\delta^{3}\left(R_{i}+r-R_{j}\right)$ in Equation (87), and thus obtain

(90) $$\rho^{2}\tilde{g}\left(r\right)=\frac{1}{V}\int \frac{d^{2}\Omega }{4\pi }\langle \underset{ij}{\sum^{\prime }}\delta^{3}\left(R_{i}+r-R_{j}\right)\underset{1}{\underbrace{\int d^{3}r_{1}\;\delta^{3}\left(r_{1}-R_{i}\right)}}\rangle =\frac{1}{V}\int \frac{d^{2}\Omega }{4\pi }\langle \underset{ij}{\sum^{\prime }}\delta^{3}\left(R_{i}+r-R_{j}\right)\rangle \;,$$

which finally leads to

(91) $$\rho \tilde{g}\left(r\right)=\int \frac{d^{2}\Omega }{4\pi }\langle \frac{1}{N}\sum_{i}\sum_{j\neq i}\delta^{3}\left(R_{i}+r-R_{j}\right)\rangle \;.$$

At zero temperature, we can neglect the average and simply write

(92) $$\rho \tilde{g}\left(r\right)=\int \frac{d^{2}\Omega }{4\pi }\frac{1}{N}\sum_{i}\sum_{j\neq i}\delta^{3}\left(R_{i}+r-R_{j}\right)=\int \frac{d^{2}\Omega }{4\pi }\sum_{R\neq 0}\delta^{3}\left(r-R\right)=\sum_{R\neq 0}\frac{1}{4\pi R^{2}}\delta \left(r-R\right)\;,$$

where in the last step we have followed the same path leading to Equation (41).

We can similarly proceed for the functions at Equations (31) and (35), which can be computed by the following formulae:

(93) $${\tilde{g}}_{0}\left(r\right)\approx \frac{1}{\rho^{2}V}\sum_{\alpha =1}^{M}v_{c}\frac{1}{N_{\gamma }}\sum_{|\gamma |=r}\frac{\langle c_{\alpha }\rangle }{v_{c}}\frac{\langle c_{\alpha +\gamma }\rangle }{v_{c}}=\frac{1}{M\rho^{2}v_{c}^{2}}\sum_{\alpha =1}^{M}\langle c_{\alpha }\rangle \frac{1}{N_{\gamma }}\sum_{|\gamma |=r}\langle c_{\alpha +\gamma }\rangle$$

and

(94) $$\tilde{h}\left(r\right)\approx \frac{1}{M\rho^{2}v_{c}^{2}}\sum_{\alpha =1}^{M}\frac{1}{N_{\gamma }}\sum_{|\gamma |=r}\langle c_{\alpha }c_{\alpha +\gamma }\rangle \ln\frac{\langle c_{\alpha }c_{\alpha +\gamma }\rangle }{\langle c_{\alpha }\rangle \langle c_{\alpha +\gamma }\rangle }\;.$$

While $\tilde{g}\left(r\right)$ is the statistical average of an estimator whose histogram can be updated in the course of the simulation (see Equation (86)), ${\tilde{g}}_{0}\left(r\right)$ can only be estimated at the end of simulation, once $\langle c_{\alpha }\rangle$ has been evaluated for every α with effort comparable to that made for the one-body entropy. Much more costly is the calculation of $\tilde{h}\left(r\right)$, which should also be performed at the end of simulation after evaluating $\langle c_{\alpha }c_{\beta }\rangle$ for every α and β.

Using translational lattice symmetry, the radial distribution functions and $\tilde{h}\left(r\right)$ of a crystal can also be written as:

(95) $$\begin{array}{ccc} \rho \tilde{g}\left(r\right) & = & \int_{C}d^{3}r_{1}\int \frac{d^{2}\Omega }{4\pi }\;\rho^{\left(2\right)}\left(r_{1},r_{1}+r\right)\;;\;\;\;\;\;\;\rho {\tilde{g}}_{0}\left(r\right)=\int_{C}d^{3}r_{1}\int \frac{d^{2}\Omega }{4\pi }\;\rho^{\left(1\right)}\left(r_{1}\right)\rho^{\left(1\right)}\left(r_{1}+r\right)\;;\\ \rho \tilde{h}\left(r\right) & = & \int_{C}d^{3}r_{1}\int \frac{d^{2}\Omega }{4\pi }\;\rho^{\left(2\right)}\left(r_{1},r_{1}+r\right)\ln\frac{\rho^{\left(2\right)}\left(r_{1},r_{1}+r\right)}{\rho^{\left(1\right)}\left(r_{1}\right)\rho^{\left(1\right)}\left(r_{1}+r\right)}\;, \end{array}$$

leading to simplifying Equations (85), (93), and (94) into

(96) $$\begin{array}{ccc} \tilde{g}\left(r\right) & = & \langle \sum_{\alpha =1}^{M/N}\delta_{c_{\alpha },1}\frac{\sum_{|\gamma |=r}c_{\alpha +\gamma }}{4\pi r^{2}\Delta r\rho }\rangle \;;\;\;\;\;\;\;{\tilde{g}}_{0}\left(r\right)=\sum_{\alpha =1}^{M/N}\langle c_{\alpha }\rangle \frac{\sum_{|\gamma |=r}\langle c_{\alpha +\gamma }\rangle }{4\pi r^{2}\Delta r\rho }\;;\\ \tilde{h}\left(r\right) & = & \sum_{\alpha =1}^{M/N}\frac{1}{4\pi r^{2}\Delta r\rho }\sum_{|\gamma |=r}\langle c_{\alpha }c_{\alpha +\gamma }\rangle \ln\frac{\langle c_{\alpha }c_{\alpha +\gamma }\rangle }{\langle c_{\alpha }\rangle \langle c_{\alpha +\gamma }\rangle }\;. \end{array}$$

In the above formulae, the α index only runs over the cubes contained in a Wigner–Seitz/Voronoi cell of the lattice, while the β sum is still carried out over all cubes in the simulation box.

### 3.7. Numerical Tests

We first examine the shape of the structure functions $\tilde{g}\left(r\right)$ and ${\tilde{g}}_{0}\left(r\right)$ for hard spheres, choosing a *r* resolution of Δr=0.05 (in units of the particle diameter σ). We take a system of N=4000 particles arranged in a fcc lattice with packing fraction η=0.600 (recall that the melting value is approximately 0.545). Periodic conditions are applied at the system boundary. In order to constrain the crystal in space, we keep one particle fixed during the simulation. As for ${\tilde{g}}_{0}\left(r\right)$, we employ the Tarazona ansatz for α=95 (see Equation (39)), a value providing the best fit to the one-body density drawn from simulation.

**Figure 1:**
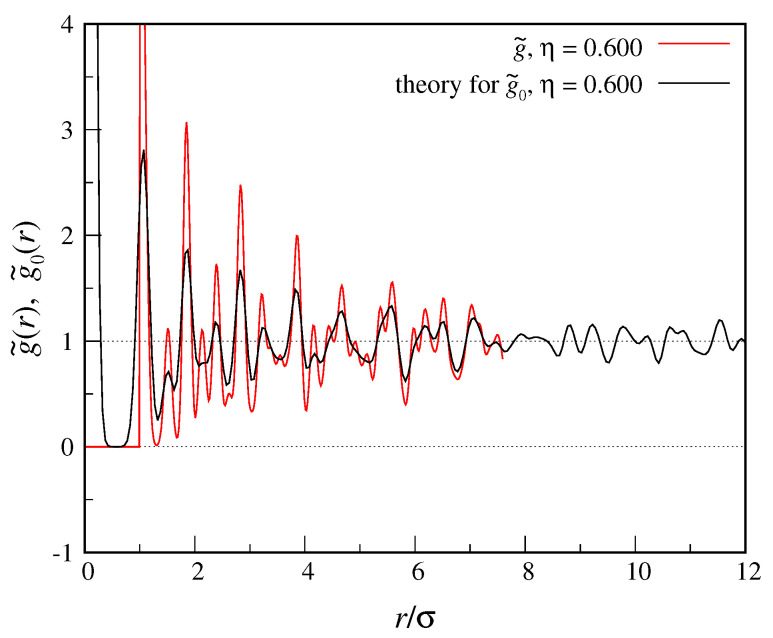
We show a comparison between $\tilde{g}\left(r\right)$ for a fcc crystal of hard spheres (η=0.600) and the ${\tilde{g}}_{0}\left(r\right)$ function given in Equation (39), where the value of α (95) has been chosen such that the Tarazona ansatz (21) fits at best the one-body density drawn from simulation.

We use the standard Metropolis Monte Carlo (MC) algorithm, constantly adjusting the maximum shift of a particle during equilibration until the fraction of accepted moves becomes close to 50% (then, the maximum shift is no longer changed). We produce 50,000 MC cycles in the equilibration run, whereas CE averages are computed over a total of further $2\times 10^{5}$ cycles. Our results are plotted in Figure 1. While at short distances $\tilde{g}\left(r\right)$ and ${\tilde{g}}_{0}\left(r\right)$ are rather different, as *r* increases the oscillations of the two functions become closer and closer in amplitude.

To obtain the one-body density with sufficient accuracy, we use a grid of about 50 points along each space direction in the unit cell. However, this grid resolution is too high for allowing the computation of $\tilde{h}\left(r\right)$, as the memory requirements for processing the $\langle c_{\alpha }c_{\beta }\rangle$ data are very huge. On the other hand, a coarser grid is incompatible with the Δr chosen.

To get closer to achieving our goal, i.e., to ascertain the *N* dependence of the two-body entropy for a crystal, we consider a two-dimensional system—hard disks. For this system, the transformation from fluid to solid occurs in two stages, via an intermediate hexatic fluid phase [36] (the transition from isotropic to hexatic fluid is first-order, whereas the hexatic-solid transition is continuous and occurs at η=0.700). We consider a system of N=1152 hard disks, arranged in a triangular crystal with packing fraction η=0.800, and a mesh consisting of about 80 points along each direction in the unit cell. Even though translational correlations are only quasi-long-ranged in an infinite two-dimensional crystal, when one of the particles is kept artificially fixed this specificity is lost and the (finite) two-dimensional crystal is made fully similar to a three-dimensional crystal. Observe also that an infinite two-dimensional crystal shares at least the same breaking of rotational symmetry typical of an infinite three-dimensional crystal.

**Figure 2:**
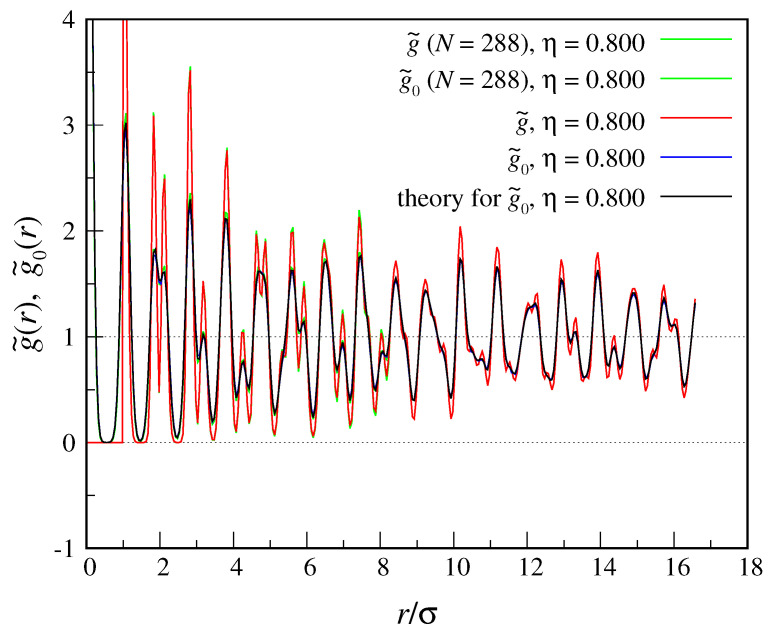
Structure functions $\tilde{g}\left(r\right)$ and ${\tilde{g}}_{0}\left(r\right)$ for a triangular crystal of hard disks (η=0.800). We report data for two sizes, N=288 and N=1152. For comparison, we also plot the ${\tilde{g}}_{0}\left(r\right)$ function in Equation (40) for α=75. As is clear, the Tarazona ansatz represents an excellent model for the one-body density of the weakly-constrained hard-disk crystal.

As before, we first look at the structure functions drawn from simulation, $\tilde{g}\left(r\right)$ and ${\tilde{g}}_{0}\left(r\right)$. Our results are plotted in Figure 2, together with the ${\tilde{g}}_{0}\left(r\right)$ function of Equation (40) for α=75. For this α the matching between the two ${\tilde{g}}_{0}$ functions is nearly perfect, indicating that the peaks of the one-body density are (to a high level of accuracy) Gaussian in shape. For η=0.800 we find $S_{1}/N=-2.156$.

**Figure 3:**
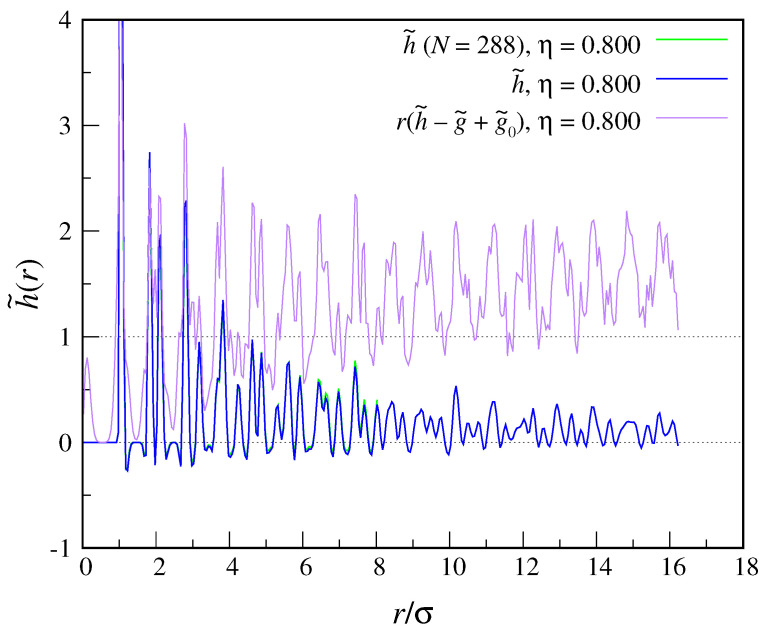
The function $\tilde{h}\left(r\right)$ for hard disks (η=0.800). As in Figure 2, data for two sizes are shown, namely, N=288 and N=1152. It appears that the oscillations of $\tilde{h}\left(r\right)$ decay very slowly, which implies slow convergence of the integrand in Equation (36) to zero.

In Figure 3, we show our main result, $\tilde{h}\left(r\right)$, for η=0.800 and two different crystal sizes, N=288 and 1152. We point out that, in order to obtain these data, we had to run a separate simulation for each *r*, as the memory usage is rather extreme. To be sure, we have computed the ${\tilde{g}}_{0}$ values in an independent way, i.e., using the same program loop written for $\tilde{h}\left(r\right)$, eventually finding the same results as in Figure 2. Looking at Figure 3, we see that $\tilde{h}\left(r\right)$ shows a series of peaks at neighbor positions and in the valleys within, taking preferentially positive values (meaning that its oscillations are not centered around zero). However, the damping of large-distance oscillations is too gradual to allow us to assess the nature of the asymptotic decay of $\tilde{h}\left(r\right)$ and then compute $S_{2}$. We must attempt a few explanations for this behavior of $\tilde{h}\left(r\right)$: On one hand, the decay of $\tilde{h}\left(r\right)$ may really be slow (at least in two dimensions), but $S_{2}$ would nonetheless be extensive, which implies a large $S_{2}/N$ value. It may as well be that constraining the crystal in space by hinging the position of one particle has a strong effect on the speed of $\tilde{h}$ decay, which only a finite-size scaling of data can relieve. Indeed, when going from N=288 to N=1152 the values of $\tilde{h}$ are slightly shifted downwards.

In summary, we have not reached any clear demonstration of $S_{2}$ extensivity in a crystal. This task has proved to be very hard to settle numerically. Our hope is that, based on our preparatory work, other authors with more powerful computational resources at their disposal can push the numerical analysis forward and eventually come up with a definite solution of the problem.

## 4. Conclusions

In this paper, we inquired into the possibility of extending the zero-RMPE criterion, a popular one-phase criterion of freezing for simple fluids, to also cover the melting of a solid. After revisiting the derivation of the entropy MPCE in the canonical ensemble, we argued that the formula applies for a crystal too. We exploited lattice symmetries to constrain the structures of one- and two-body densities, so as to gain as much information as possible on the first few terms in the entropy expansion. While this was enough to prove that the crystal one-body entropy is an extensive quantity, the information obtained was not sufficient to hold the same for the two-body entropy, whose scaling with the size of the crystal remains elusive. We thus attempted to clarify the question numerically, but we faced an insurmountable obstacle in the computational and memory limitations. To alleviate the problem, we turned towards a two-dimensional case, namely, hard disks, but with poor results: the structure function that must be integrated over distances to obtain the two-body entropy is weakly convergent to zero. In the near future, we plan to check whether the situation is more favorable for a different two-dimensional interaction, either endowed with an attractive tail (e.g., the Lennard-Jones potential) or provided with a soft core (for example, a Gaussian repulsion).

## Appendix A. Truncating the Entropy Expansion

We hereafter give an interpretation of the successive estimates of $S_{N}^{\mathrm{exc}}/k_{B}=-\langle \ln P_{1\ldots N}\rangle$ obtained by stopping the expansion (13) at a given order of correlations. We show that each truncated entropy expansion can be arranged in the form $-\langle \ln P_{1\ldots N}^{⋆}\rangle$, where $P_{1\ldots N}^{⋆}$ is a functional of all the MDFs up to *n*-th order, for n=1,2,…,N (however, without claiming that $P_{1\ldots N}^{⋆}$ represents a proper, i.e., normalized distribution). Our method resembles the one originally devised by H. S. Green to express the canonical entropy of a *N*-particle fluid in terms of correlation functions [1]. While H. S. Green correctly inferred the first three terms in the expansion, he did not provide a general recipe to obtain the further terms recursively.

For N=1 there is only one MDF, $P_{1}\equiv P^{\left(1\right)}\left(r_{1}\right)$, in terms of which a fully symmetric approximation to $P_{1\ldots N}$ can be constructed:

(A1) $$1\mathrm{st}-\mathrm{order}\;\;\mathrm{approximation}:\;P_{1\ldots N}^{⋆}=\prod_{i}^{N}P_{i}\;.$$

Notice that $-\langle \ln P_{1\ldots N}^{⋆}\rangle =S_{N}^{\left(1\right)}/k_{B}$.

To obtain a better approximation we consider a system of two particles. Since

(A2) $$P_{12}=P_{1}P_{2}\times \frac{P_{12}}{P_{1}P_{2}}\;,$$

we see that $P_{12}$ is the product of the 1st-order approximation (A1) times a correction factor $P_{12}/\left(P_{1}P_{2}\right)=Q_{12}$. Assuming that in a *N*-particle system each distinct pair of particles contributes the same factor to $P_{1\ldots N}^{⋆}$, we arrive at the

(A3) $$2\mathrm{nd}-\mathrm{order}\;\;\mathrm{approximation}:\;P_{1\ldots N}^{⋆}=\prod_{i}^{N}P_{i}\prod_{i<j}^{N}Q_{ij}\;.$$

The number of factors in the second product is N(N−1)/2. For this $P_{1\ldots N}^{⋆}$ we obtain

(A4) −〈lnP1…N⋆〉=−N∫P1lnP1−N2∫P12lnQ12=SN(2)/kB.

Moving to N=3, we observe that

(A5) $$P_{123}=P_{1}P_{2}P_{3}Q_{12}Q_{13}Q_{23}\times \frac{Q_{123}}{Q_{12}Q_{13}Q_{23}}\;,$$

which is the second-order approximation to $P_{123}^{⋆}$ times a correction factor. In the event that each distinct triplet of particles contributes the same factor to $P_{1\ldots N}^{⋆}$, we obtain the

(A6) $$3\mathrm{rd}-\mathrm{order}\;\;\mathrm{approximation}:\;P_{1\ldots N}^{⋆}=\prod_{i}^{N}P_{i}\prod_{i<j}^{N}Q_{ij}\prod_{i<j<k}^{N}\frac{Q_{ijk}}{Q_{ij}Q_{ik}Q_{jk}}\;.$$

Notice that a different expression for the latter ratio is

(A7) $$\frac{Q_{ijk}}{Q_{ij}Q_{ik}Q_{jk}}=\frac{P_{ijk}}{\frac{P_{ij}P_{ik}P_{jk}}{P_{i}P_{j}P_{k}}}\;.$$

The number of factors in the third product is N(N−1)(N−2)/6. For this $P_{1\ldots N}^{⋆}$, the approximate entropy is

(A8) −〈lnP1…N⋆〉=−N∫P1lnP1−N2∫P12lnQ12−N3∫P123lnQ123−32∫P12lnQ12=SN(3)kB.

We can similarly proceed to derive higher-order approximations. The 4-body MDF of a system of N=4 particles is trivially decomposed as

(A9) $$\begin{array}{ccc} P_{1234} & = & P_{1}P_{2}P_{3}P_{4}Q_{12}Q_{13}Q_{14}Q_{23}Q_{24}Q_{34}\frac{Q_{123}}{Q_{12}Q_{13}Q_{23}}\frac{Q_{124}}{Q_{12}Q_{14}Q_{24}}\frac{Q_{134}}{Q_{13}Q_{14}Q_{34}}\frac{Q_{234}}{Q_{23}Q_{24}Q_{34}}\\ & \times & \frac{Q_{1234}}{Q_{12}Q_{13}Q_{14}Q_{23}Q_{24}Q_{34}\frac{Q_{123}}{Q_{12}Q_{13}Q_{23}}\frac{Q_{124}}{Q_{12}Q_{14}Q_{24}}\frac{Q_{134}}{Q_{13}Q_{14}Q_{34}}\frac{Q_{234}}{Q_{23}Q_{24}Q_{34}}}\\ & = & P_{1}P_{2}P_{3}P_{4}Q_{12}Q_{13}Q_{14}Q_{23}Q_{24}Q_{34}\frac{Q_{123}}{Q_{12}Q_{13}Q_{23}}\frac{Q_{124}}{Q_{12}Q_{14}Q_{24}}\frac{Q_{134}}{Q_{13}Q_{14}Q_{34}}\frac{Q_{234}}{Q_{23}Q_{24}Q_{34}}\\ & \times & \frac{Q_{1234}}{\frac{Q_{123}Q_{124}Q_{134}Q_{234}}{Q_{12}Q_{13}Q_{14}Q_{23}Q_{24}Q_{34}}}\;, \end{array}$$

whence the

(A10) $$4\mathrm{th}-\mathrm{order}\;\;\mathrm{approximation}:\;P_{1\ldots N}^{⋆}=\prod_{i}^{N}P_{i}\prod_{i<j}^{N}Q_{ij}\prod_{i<j<k}^{N}\frac{Q_{ijk}}{Q_{ij}Q_{ik}Q_{jk}}\prod_{i<j<k<l}^{N}\frac{Q_{ijkl}}{\frac{Q_{ijk}Q_{ijl}Q_{ikl}Q_{jkl}}{Q_{ij}Q_{ik}Q_{il}Q_{jk}Q_{jl}Q_{kl}}}\;.$$

Notice that a different expression for the latter ratio is

(A11) $$\frac{Q_{ijkl}}{\frac{Q_{ijk}Q_{ijl}Q_{ikl}Q_{jkl}}{Q_{ij}Q_{ik}Q_{il}Q_{jk}Q_{jl}Q_{kl}}}=\frac{P_{ijkl}}{\frac{P_{ijk}P_{ijl}P_{ikl}P_{jkl}}{\frac{P_{ij}P_{ik}P_{il}P_{jk}P_{jl}P_{kl}}{P_{i}P_{j}P_{k}P_{l}}}}\;.$$

For this $P_{1\ldots N}^{⋆}$ we obtain

(A12) −〈lnP1…N⋆〉=−N∫P1lnP1−N2∫P12lnQ12−N3∫P123lnQ123−32∫P12lnQ12−N4∫P1234lnQ1234−43∫P123lnQ123+42∫P12lnQ12=SN(4)/kB.

Eventually, with the last *N*th-order approximation we recover the exact distribution, namely, $P_{1\ldots N}^{⋆}=P_{1\ldots N}$, and the full entropy. Notice that, except for n=1 and *N*, the *n*th-order functional $P_{1\ldots N}^{⋆}$ is not normalized.

We now provide a formalization of the procedure sketched above. For each value of *n* and each grouping $I_{n}=\left\{i_{1},i_{2},\ldots i_{n}\right\}$ of *n* particle indices, we write $P\left(I_{n}\right)\equiv P_{i_{1}\ldots i_{n}}$ as a product of positive cumulant factors to be determined recursively, that is

(A13) $$P\left(I_{n}\right)=\prod_{S_{1}\subset I_{n}}C\left(S_{1}\right)\cdots \prod_{S_{n-1}\subset I_{n}}C\left(S_{n-1}\right)\times C\left(I_{n}\right)\;,$$

where $\prod_{S_{k}\subset I_{n}}$ indicates the product over all *k*-tuples of distinct entries from $I_{n}$—there are nk factors in the product $\prod_{S_{k}\subset I_{n}}$. As shown before, $C\left(\left\{i\right\}\right)=P_{i},C\left(\left\{i,j\right\}\right)=P_{ij}/\left(P_{i}P_{j}\right),C\left(\left\{i,j,k\right\}\right)=P_{ijk}P_{i}P_{j}P_{k}/\left(P_{ij}P_{ik}P_{jk}\right)$, and so on. Taking the logarithm of (A13) we obtain:

(A14) $$\ln P\left(I_{n}\right)=\ln C\left(I_{n}\right)+\sum_{S_{n-1}\subset I_{n}}\ln C\left(S_{n-1}\right)+\ldots +\sum_{S_{1}\subset I_{n}}\ln C\left(S_{1}\right)\;,$$

which can be solved with respect to cumulants by the Möbius inversion formula (see, e.g., Equations (3) and (4) of reference [5]):

(A15) $$\ln C\left(I_{n}\right)=\ln P\left(I_{n}\right)-\sum_{S_{n-1}\subset I_{n}}\ln P\left(S_{n-1}\right)+\ldots +{\left(-1\right)}^{n-1}\sum_{S_{1}\subset I_{n}}\ln P\left(S_{1}\right)\;,$$

which is in turn equivalent to writing

(A16) $$C\left(I_{n}\right)=\frac{P(I_{n})}{\frac{\prod_{S_{n-1}\subset I_{n}}P\left(S_{n-1}\right)}{\frac{\vdots }{\prod_{S_{1}\subset I_{n}}P\left(S_{1}\right)}}}\;.$$

Equations (A7) and (A11) are just particular cases of the above formula, respectively for n=3 and n=4. Once the cumulants have been determined, the functional $P_{1\ldots N}^{*}$ of *M*-th order (for M=1,…,N) can be written, by an obvious change of notation, as

(A17) $$P_{1\ldots N}^{*}=\prod_{i}^{N}C_{i}\prod_{i<j}^{N}C_{ij}\cdots \prod_{i_{1}<\ldots <i_{M}}^{N}C_{i_{1}\ldots i_{M}}\;,$$

leading to

(A18) −lnP1…N*=−N∫P1lnC1−N2∫P12lnC12−…−NM∫P12…MlnC12…M.

In view of Equation (A16), the above quantity is nothing but $S_{N}^{\left(M\right)}$.
